# *N*-(2-Phenyl­imidazo[1,2-*a*]pyridin-3-yl)acetamide

**DOI:** 10.1107/S1600536808011501

**Published:** 2008-04-26

**Authors:** Abderrahmane Anaflous, Hanane Albay, Nour-eddine Benchat, Brahim El Bali, Michal Dušek, Karla Fejfarová

**Affiliations:** a Département de Chimie, Faculté des Sciences, BP 717 Oujda, Morocco; b Département de Chimie, Faculté des Sciences, BP717 Oujda, Morocco; c Laboratory of Mineral Solid and Analytical Chemistry, ‘LMSAC’, Department of Chemistry, Faculty of Sciences, University Mohamed I, PO Box 717, 60000 Oujda, Morocco; d Institute of Physics, Na Slovance 2, 182 21 Praha 8, Czech Republic

## Abstract

The crystal structure of the title compound, C_15_H_13_N_3_O, consists of columns of mol­ecules that are inter­connected by N—H⋯N hydrogen bonds in the direction of the *b* axis. The torsion angle between the imidazo[1,2-*a*]pyridine ring system and the phenyl ring is 9.04 (5)°.

## Related literature

For general background, see Anaflous *et al.* (2004 ▶); Gueffier *et al.* (1998 ▶); Mavel *et al.* (2002 ▶).
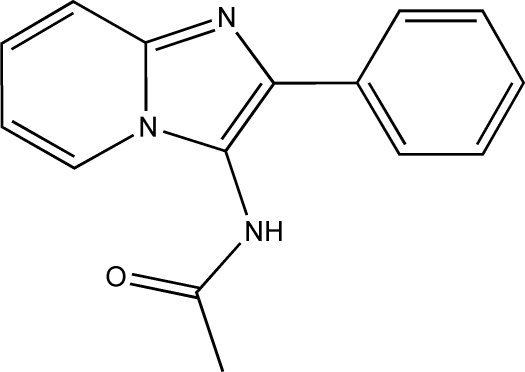


## Experimental

### 

#### Crystal data

C_15_H_13_N_3_O
*M* *_r_* = 251.3
Monoclinic, 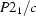

*a* = 13.9680 (5) Å
*b* = 5.6784 (2) Å
*c* = 15.8145 (5) Å
β = 101.039 (3)°
*V* = 1231.13 (7) Å^3^
*Z* = 4
Mo *K*α radiation
μ = 0.09 mm^−1^
*T* = 120 K
0.58 × 0.25 × 0.17 mm

#### Data collection

Oxford Diffraction Xcalibur2 diffractometer with Sapphire2 CCD detector
Absorption correction: none
15703 measured reflections
2556 independent reflections
1544 reflections with *I* > 3σ(*I*)
*R*_int_ = 0.054

#### Refinement

*R*[*F* ^2^ > 2σ(*F* ^2^)] = 0.036
*wR*(*F* ^2^) = 0.084
*S* = 1.00
2556 reflections
175 parameters
1 restraint
H atoms treated by a mixture of independent and constrained refinement
Δρ_max_ = 0.17 e Å^−3^
Δρ_min_ = −0.14 e Å^−3^

### 

Data collection: *CrysAlis CCD* (Oxford Diffraction, 2008 ▶); cell refinement: *CrysAlis RED* (Oxford Diffraction, 2008 ▶); data reduction: *CrysAlis RED*; program(s) used to solve structure: *SIR2002* (Burla *et al.*, 2003 ▶); program(s) used to refine structure: *JANA2006* (Petříček *et al.*, 2006 ▶); molecular graphics: *DIAMOND* (Brandenburg & Putz, 1999 ▶); software used to prepare material for publication: *JANA2006*.

## Supplementary Material

Crystal structure: contains datablocks global, I. DOI: 10.1107/S1600536808011501/fj2112sup1.cif

Structure factors: contains datablocks I. DOI: 10.1107/S1600536808011501/fj2112Isup2.hkl

Additional supplementary materials:  crystallographic information; 3D view; checkCIF report

## Figures and Tables

**Table 1:** Hydrogen-bond geometry (Å, °)

*D*—H⋯*A*	*D*—H	H⋯*A*	*D*⋯*A*	*D*—H⋯*A*
N3—H3*n*⋯N1^i^	0.880 (12)	2.162 (12)	3.0219 (16)	165.4 (13)

Symmetry code: (i) 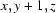.

**Fig. 1.**
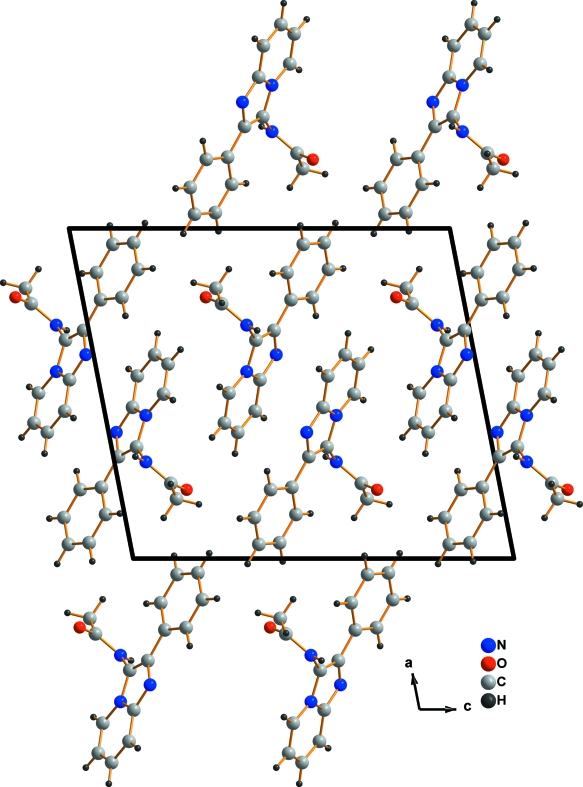
View of the unit cell of the title structure along the axis b

**Fig. 2.**
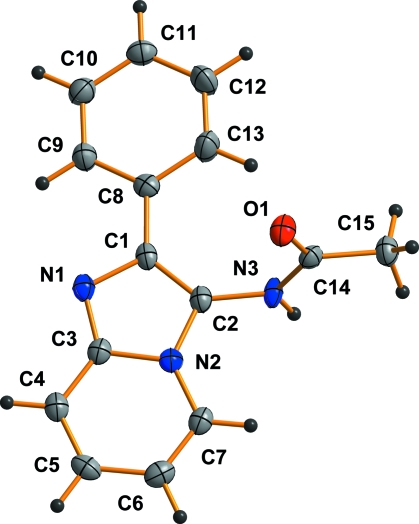
Asymetric unit of title compound, showing 50% displacement ellispoids for non-H atoms.

**Fig. 3.**
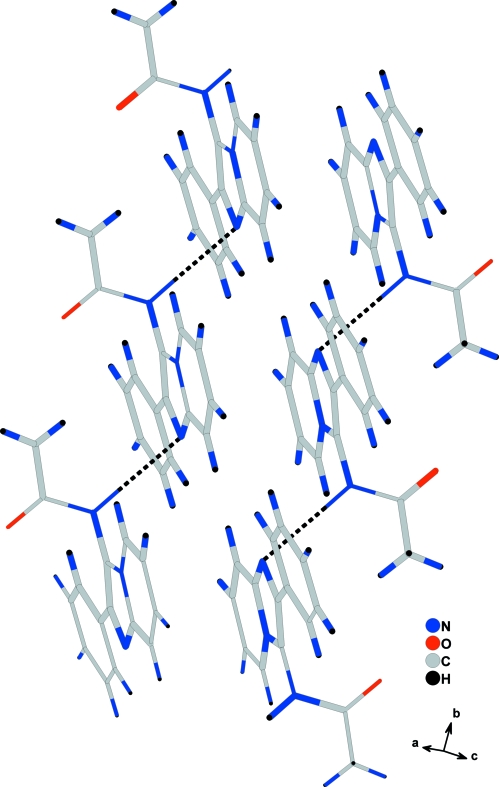
The columns of molecules showing N—H···N hydrogen bonds

## supplementary crystallographic information

### Comment

In recent years, functionalized imidazo[1,2-*a*]pyridine and imidazo[1,2-*a*]pyrimidine systems attracted persistent interest due to their biological activities (Anaflous *et al.*, 2004 and reference herein). The screening of imidazo[1,2-*a*]pyridine derivatives against tuberculosis showed interesting results (Anaflous *et al.*, 2004) and many functionalized imidazo[1,2-*a*]pyridines bearing a thioether side chain at the 3 position are reported as highly active against human cytomegalovirus and /or varicella-zoster virus (Gueffier *et al.*, 1998 & Mavel *et al.*, 2002).

We report in the present paper on the synthesis and crystal structure of *N*-(2-phenylimidazo[1,2-*a*]pyridin-3-yl)acetamide (I).

The molecules of the title compound are interconnected into columns extended along b by an N3—H3n···N1 hydrogen bonds (see Tab. 1). No bonding has been found between the columns that appear to be quite isolated.

Bonds and angles values are usual as those reported in similar compounds.

The torsion angle between the imidazo[1,2-*a*]pyridine and phenyl ring is 9.04 (5)°

### Experimental

The commercially available 2-phenylimidazo[1,2-*a*]pyridin-3-amine (0.50 g, 2.4 mmole) in toluene (10 ml, 94 mmole) was treated with acetic anhydride (0.3 ml, 3.2 mmole). The mixture was stirred for two hours. Toluene was eliminated under reduced pressure and the residue was washed with water to give, after drying, 0.45 g (1.8 mmole) of *N*-(2-phenylimidazo[1,2-*a*]pyridin-3-yl)acetamide as colorless crystals.

### Crystal data

**Table:**

C_15_H_13_N_3_O	*F*_000_ = 528
*M**_r_* = 251.3	*D*_x_ = 1.355 Mg m^−^^3^
Monoclinic, *P*2_1_/*c*	Mo *K*α radiation λ = 0.71073 Å
Hall symbol: -P 2ybc	Cell parameters from 4755 reflections
*a* = 13.9680 (5) Å	θ = 2.6–26.5º
*b* = 5.6784 (2) Å	µ = 0.09 mm^−^^1^
*c* = 15.8145 (5) Å	*T* = 120 K
β = 101.039 (3)º	Prism, colorless
*V* = 1231.13 (7) Å^3^	0.58 × 0.25 × 0.17 mm
*Z* = 4	

### Data collection

**Table:**

Oxford Diffraction Xcalibur2 diffractometer with Sapphire2 CCD detector	2556 independent reflections
Radiation source: X-ray tube	1544 reflections with *I* > 3σ(*I*)
Monochromator: graphite	*R*_int_ = 0.054
Detector resolution: 8.3438 pixels mm^-1^	θ_max_ = 26.5º
*T* = 120 K	θ_min_ = 2.6º
Rotation method data acquisition using ω scans	*h* = −17→17
Absorption correction: none	*k* = −7→7
15703 measured reflections	*l* = −19→19

### Refinement

**Table:**

Refinement on *F*^2^	45 constraints
*R*[*F*^2^ > 2σ(*F*^2^)] = 0.036	H atoms treated by a mixture of independent and constrained refinement
*wR*(*F*^2^) = 0.084	Weighting scheme based on measured s.u.'s *w* = 1/[σ^2^(*I*) + 0.0016*I*^2^]
*S* = 1.00	(Δ/σ)_max_ = 0.006
2556 reflections	Δρ_max_ = 0.17 e Å^−^^3^
175 parameters	Δρ_min_ = −0.14 e Å^−^^3^
1 restraint	Extinction correction: none

### Special details

**Table:**

Refinement. The refinement was carried out against all reflections. The conventional *R*-factor is always based on *F*. The goodness of fit as well as the weighted *R*-factor are based on *F* and *F*^2^ for refinement carried out on *F* and *F*^2^, respectively. The threshold expression is used only for calculating *R*-factors *etc*. and it is not relevant to the choice of reflections for refinement.All the H atoms were discernible in difference Fourier maps and could be refined to reasonable geometry. According to standard procedures for organic compounds the H atoms bonded to C atoms were constrained to ideal positions. The N—H distances were restrained to 0.87 Å with σ 0.01. The isotropic atomic displacement parameters of hydrogen atoms were evaluated as 1.2**U*_eq_ of the parent atom.The program used for refinement, Jana2006, uses the weighting scheme based on the experimental expectations, see _refine_ls_weighting_details, that does not force *S* to be one. Therefore the values of *S* are usually larger than the ones from the *SHELX* program.

### Fractional atomic coordinates and isotropic or equivalent isotropic displacement parameters (Å^2^)

**Table:**

	*x*	*y*	*z*	*U*_iso_*/*U*_eq_	
N1	0.61764 (8)	−0.2561 (2)	0.47985 (7)	0.0230 (4)	
N2	0.56626 (7)	0.05089 (18)	0.39364 (7)	0.0206 (4)	
N3	0.70718 (11)	0.3084 (2)	0.41732 (9)	0.0235 (5)	
O1	0.79160 (9)	0.1417 (2)	0.32433 (8)	0.0298 (4)	
C1	0.69090 (11)	−0.0912 (2)	0.48648 (9)	0.0210 (5)	
C2	0.66186 (9)	0.09842 (19)	0.43398 (8)	0.0224 (4)	
C3	0.54173 (10)	−0.1658 (2)	0.42431 (9)	0.0211 (5)	
C4	0.44673 (11)	−0.2519 (3)	0.39578 (9)	0.0238 (5)	
C5	0.38234 (11)	−0.1217 (3)	0.33930 (9)	0.0262 (5)	
C6	0.41070 (11)	0.0979 (2)	0.30893 (9)	0.0272 (5)	
C7	0.50162 (10)	0.1807 (3)	0.33604 (9)	0.0233 (5)	
C8	0.78417 (11)	−0.1346 (2)	0.54688 (10)	0.0220 (6)	
C9	0.79074 (12)	−0.3221 (3)	0.60385 (10)	0.0285 (5)	
C10	0.87592 (12)	−0.3699 (3)	0.66086 (10)	0.0317 (5)	
C11	0.95689 (11)	−0.2316 (3)	0.66266 (10)	0.0290 (5)	
C12	0.95210 (12)	−0.0434 (3)	0.60733 (10)	0.0373 (6)	
C13	0.86634 (12)	0.0062 (3)	0.54974 (11)	0.0356 (6)	
C14	0.77211 (11)	0.3171 (3)	0.36247 (10)	0.0220 (5)	
C15	0.81873 (12)	0.5518 (3)	0.35553 (11)	0.0294 (6)	
H3n	0.6911 (10)	0.4370 (19)	0.4423 (9)	0.0282*	
H4	0.42756	−0.400812	0.415958	0.0285*	
H5	0.317152	−0.17869	0.319756	0.0314*	
H6	0.364663	0.187888	0.268776	0.0327*	
H7	0.520896	0.328874	0.315284	0.028*	
H9	0.734759	−0.420445	0.603502	0.0341*	
H10	0.878784	−0.500882	0.699648	0.038*	
H11	1.016393	−0.265961	0.702246	0.0348*	
H12	1.008436	0.054304	0.608524	0.0447*	
H13	0.863646	0.13862	0.511608	0.0427*	
H15a	0.77381	0.67451	0.36305	0.0353*	
H15b	0.876474	0.564915	0.399312	0.0353*	
H15c	0.8358 (10)	0.5666 (19)	0.2998 (9)	0.0353*	

### Atomic displacement parameters (Å^2^)

**Table:**

	*U*^11^	*U*^22^	*U*^33^	*U*^12^	*U*^13^	*U*^23^
N1	0.0273 (7)	0.0188 (6)	0.0250 (7)	−0.0018 (5)	0.0100 (6)	−0.0017 (5)
N2	0.0230 (7)	0.0198 (6)	0.0205 (6)	0.0005 (5)	0.0079 (5)	−0.0023 (5)
N3	0.0343 (9)	0.0126 (7)	0.0271 (8)	−0.0014 (7)	0.0148 (7)	−0.0020 (7)
O1	0.0405 (7)	0.0228 (6)	0.0305 (7)	0.0012 (6)	0.0176 (6)	−0.0026 (5)
C1	0.0263 (9)	0.0171 (7)	0.0225 (8)	−0.0029 (7)	0.0119 (7)	−0.0042 (6)
C2	0.0267 (8)	0.0183 (6)	0.0245 (7)	−0.0015 (6)	0.0108 (6)	−0.0028 (5)
C3	0.0275 (9)	0.0186 (7)	0.0192 (8)	0.0014 (7)	0.0098 (7)	−0.0021 (6)
C4	0.0304 (9)	0.0210 (8)	0.0227 (8)	−0.0023 (7)	0.0120 (7)	−0.0040 (6)
C5	0.0217 (9)	0.0333 (8)	0.0247 (8)	−0.0023 (7)	0.0074 (7)	−0.0094 (7)
C6	0.0317 (8)	0.0309 (8)	0.0195 (8)	0.0080 (6)	0.0062 (7)	0.0003 (6)
C7	0.0319 (8)	0.0197 (8)	0.0207 (8)	0.0027 (7)	0.0112 (7)	−0.0005 (6)
C8	0.0226 (10)	0.0222 (8)	0.0222 (9)	0.0012 (7)	0.0068 (8)	−0.0058 (7)
C9	0.0296 (9)	0.0261 (9)	0.0308 (9)	−0.0033 (8)	0.0086 (7)	0.0015 (7)
C10	0.0338 (9)	0.0285 (8)	0.0327 (9)	0.0036 (7)	0.0061 (8)	0.0048 (7)
C11	0.0282 (8)	0.0324 (9)	0.0258 (8)	0.0039 (7)	0.0034 (7)	−0.0015 (7)
C12	0.0293 (10)	0.0377 (9)	0.0425 (10)	−0.0114 (8)	0.0013 (8)	0.0022 (7)
C13	0.0394 (10)	0.0306 (9)	0.0357 (10)	−0.0038 (8)	0.0040 (8)	0.0111 (8)
C14	0.0251 (10)	0.0210 (9)	0.0203 (8)	0.0030 (7)	0.0055 (7)	0.0034 (7)
C15	0.0335 (10)	0.0248 (10)	0.0331 (11)	−0.0032 (8)	0.0144 (9)	0.0020 (9)

### Geometric parameters (Å, °)

**Table:**

N1—C1	1.3763 (18)	C6—H6	0.96
N1—C3	1.3424 (17)	C7—H7	0.96
N2—C2	1.3916 (15)	C8—C9	1.387 (2)
N2—C3	1.3894 (17)	C8—C13	1.393 (2)
N2—C7	1.3689 (16)	C9—C10	1.375 (2)
N3—C2	1.3984 (19)	C9—H9	0.96
N3—C14	1.371 (2)	C10—C11	1.373 (2)
N3—H3n	0.880 (12)	C10—H10	0.96
O1—C14	1.222 (2)	C11—C12	1.375 (2)
C1—C2	1.3727 (18)	C11—H11	0.96
C1—C8	1.481 (2)	C12—C13	1.388 (2)
C3—C4	1.405 (2)	C12—H12	0.96
C4—C5	1.359 (2)	C13—H13	0.96
C4—H4	0.96	C14—C15	1.497 (2)
C5—C6	1.419 (2)	C15—H15a	0.96
C5—H5	0.96	C15—H15b	0.96
C6—C7	1.345 (2)	C15—H15c	0.960 (15)
			
C1—N1—C3	105.82 (11)	C1—C8—C9	119.17 (14)
C2—N2—C3	106.91 (10)	C1—C8—C13	122.90 (14)
C2—N2—C7	130.80 (11)	C9—C8—C13	117.93 (14)
C3—N2—C7	122.27 (11)	C8—C9—C10	121.26 (15)
C2—N3—C14	121.89 (13)	C8—C9—H9	119.372
C2—N3—H3n	117.3 (9)	C10—C9—H9	119.371
C14—N3—H3n	120.8 (9)	C9—C10—C11	120.43 (14)
N1—C1—C2	110.99 (11)	C9—C10—H10	119.784
N1—C1—C8	119.03 (12)	C11—C10—H10	119.784
C2—C1—C8	129.96 (13)	C10—C11—C12	119.48 (13)
N2—C2—N3	120.55 (11)	C10—C11—H11	120.26
N2—C2—C1	105.77 (11)	C12—C11—H11	120.259
N3—C2—C1	133.68 (12)	C11—C12—C13	120.42 (15)
N1—C3—N2	110.49 (11)	C11—C12—H12	119.792
N1—C3—C4	131.02 (13)	C13—C12—H12	119.792
N2—C3—C4	118.48 (12)	C8—C13—C12	120.48 (15)
C3—C4—C5	119.18 (14)	C8—C13—H13	119.758
C3—C4—H4	120.41	C12—C13—H13	119.758
C5—C4—H4	120.41	N3—C14—O1	121.32 (15)
C4—C5—C6	120.52 (13)	N3—C14—C15	115.37 (14)
C4—C5—H5	119.739	O1—C14—C15	123.28 (16)
C6—C5—H5	119.74	C14—C15—H15a	109.471
C5—C6—C7	120.46 (13)	C14—C15—H15b	109.472
C5—C6—H6	119.768	C14—C15—H15c	109.5 (7)
C7—C6—H6	119.769	H15a—C15—H15b	109.472
N2—C7—C6	119.07 (13)	H15a—C15—H15c	109.471
N2—C7—H7	120.466	H15b—C15—H15c	109.47
C6—C7—H7	120.465		

### Hydrogen-bond geometry (Å, °)

**Table:**

*D*—H···*A*	*D*—H	H···*A*	*D*···*A*	*D*—H···*A*
N3—H3n···N1^i^	0.880 (12)	2.162 (12)	3.0219 (16)	165.4 (13)

Symmetry codes: (i) *x*, *y*+1, *z*.
